# Understanding Immune Thrombocytopenia: Looking Out of the Box

**DOI:** 10.3389/fmed.2021.613192

**Published:** 2021-06-24

**Authors:** Alexandra Schifferli, Franco Cavalli, Bertrand Godeau, Howard A. Liebman, Mike Recher, Paul Imbach, Thomas Kühne

**Affiliations:** ^1^Department of Hematology/Oncology, University Children's Hospital Basel, Basel, Switzerland; ^2^Intercontinental Cooperative Immune thrombocytopenia (ITP) Study Group, Basel, Switzerland; ^3^Lymphoma Unit, Oncology Institute of Southern Switzerland, Bellinzona, Switzerland; ^4^Centre de Référence des Cytopénies Auto-Immunes de l'Adulte, Service de Médecine Interne, CHU Henri Mondor, AP-HP, Université Paris-Est Créteil, Créteil, France; ^5^Jane Anne Nohl Division of Hematology, Department of Medicine, Keck School of Medicine, University of Southern California, Los Angeles, CA, United States; ^6^Medical Outpatient Clinic and Immunodeficiency Laboratory, Department of Biomedicine, University Hospital and University Basel, Basel, Switzerland

**Keywords:** immune thrombocytopenia, ITP syndrome, secondary ITP, autoimmunity, immunodeficiency, cancer, overlap syndromes

## Abstract

The pathogenesis of immune thrombocytopenia (ITP) is increasingly being elucidated, and its etiology is becoming more frequently identified, leading to a diagnostic shift from primary to secondary ITP. The overlap between autoimmunity, immunodeficiency, and cancer is evident, implying more interdisciplinarity in daily care. This mini-review is based on an expert meeting on ITP organized by the Intercontinental Cooperative ITP Study Group and presents the challenges of hematologists in understanding and investigating “out of the box” concepts associated with ITP.

## Introduction

The triennial Intercontinental Cooperative ITP Study Group (ICIS) expert meeting on immune thrombocytopenia (ITP) was held for the sixth time in September 2019 in Locarno, Switzerland, where scientists and clinicians of pediatric and adult hematology, oncology, immunology, and genetics shared their ideas, experiences, and research findings to critically assess open questions and launch future joint projects (www.itpbasel.ch).

In the present mini-review, we discuss current approaches to understand ITP, following the paradigm “looking out of the box,” which aim to summarize some analogies and bridges between autoimmunity, infection, immunodeficiency, and cancer. It is without a doubt that the comprehension of pathophysiological overlap condition will help develop new targeted therapies.

## The ITP Syndrome

ITP is an autoimmune bleeding disorder caused by various etiologies, which is characterized by increased platelet destruction and impaired production, resulting in a decreased platelet count. Primary ITP is idiopathic, whereas secondary ITP is linked to an underlying condition (1). The rate of secondary ITP in children has not been studied in detail but is assumed to be rare (2.4%) (2, 3). In adults, ~18–38% of ITP patients have an underlying disease, comorbid condition, and/or comedication, making the diagnosis of secondary ITP more probable (2, 4). Secondary ITP is known to be caused by systemic autoimmune disorders, primary or secondary immunodeficiency, infectious diseases, paraneoplastic syndromes (e.g., lymphomas and other malignancies), and drug-dependent antibodies (5).

Understanding, diagnosing, and treating patients with ITP require expertise in the rapidly growing field of autoimmune disorders. Indeed, there has been an increase in ITP knowledge in recent years (6), and the number of patients with primary ITP as an idiopathic hematological disorder is gradually decreasing. However, clinical research and patient care should continue to embrace both primary and secondary ITP because of differential diagnostic considerations and a holistic understanding of it.

Cines et al. (4) first used the term “ITP syndrome” in 2009 in their review paper. The “syndrome” is characterized by both the heterogeneity of pathogenesis and the variability in clinical courses, which is a hallmark of ITP, regardless of whether it is secondary or primary. In this cited review, Cines et al. presented the hypothesis that the level of the dysfunctional immune tolerance (central vs. peripheral) predicts the treatment response and clinical course. A mistake in tolerance induction during a normal germinal center response may explain the forms of disease that are cured by steroids or rituximab. In contrast, central tolerance defects may be less responsive to immunomodulation because a large proportion of the primary repertoire is autoreactive and may be reconstituted rapidly after therapy. This was also shown in an analysis of three ITP patients relapsing after splenectomy, as the expected reduction of the B-cell memory pool after removal of the spleen was absent, whereas a persisting intrinsic B-cell hyperfunction was measured (7).

In accordance to this assumption, infection-triggered ITP (e.g., childhood ITP) exhibits the best prognosis, whereas genetically determined immune deficiency-associated ITP exhibits a rather refractory course. However, additional factors such as environmental and genetic, age, concomitant disease, and medication may impact the clinical picture via variability in platelet turnover, propensity to bleed, risk of chronic disease, and response to ITP-directed therapy.

## Autoimmunity Overlap Syndromes

ITP in patients with a systemic autoimmune disease, such as systemic lupus erythematosus or Sjögren syndrome, are evidently considered secondary ITP. Some other disease associations reveal a possible correlation or interaction, but this is yet to be clarified, for example, the one between autoimmune thyroid disorders and ITP. Hashimoto thyroiditis and Graves' disease are among the most common autoimmune disorders, and their association with ITP has extensively been discussed. The time intervals between the diagnosis of thyroid disease and that of ITP may range from months to years, still thyroid antibodies have been detected in 18–39% of ITP patients and overt hyperthyroidism in 7–8% (8–11). In children the prevalence of antithyroid antibodies was found to be significantly higher in chronic ITP (11.6%) than in the pediatric population (1.2%) (12). However, in children autoimmune thyroiditis does not seem to play a role as a prognostic factor of chronicity. Surprisingly, case reports have shown that ITP with hyperthyroidism leads to treatment-refractory thrombocytopenia (13, 14). Therefore, it remains unclear whether these underlying conditions or factors influence the phenotype and clinical course of ITP. Alternatively, these conditions can be postulated to reflect an underlying immune dysfunction, resulting in the loss of self-tolerance. When distinguishing secondary from primary ITP, there may be a thin line of separation.

## Infection and Autoimmunity

Infectious diseases and vaccinations may trigger autoimmunity, which may be true for primary ITP of childhood and for ITP associated with infections caused by Helicobacter pylori, varicella zoster, human immunodeficiency virus (HIV), and hepatitis C virus as well as for the combined pediatric vaccine against measles, rubella and mumps (5, 6, 15). By contrast, vaccination and ITP in adults have not yet been proven to be correlated, and some studies have even suggested that influenza immunization is potentially protective against autoimmunity (16). The pathogenesis appears to be multifactorial in all cases. Molecular mimicry with the cross-reaction of antiviral antibodies and platelet surface glycoproteins is widely accepted. Other theories, including the production of platelets expressing viral antigens, binding of immune complexes, and formation of autoantibodies via epitope spread, have been discussed and studied. These phenomena can result in immune system dysregulation, leading to the clonal expansion of autoreactive B-/T-cell subsets or the disturbed balance between activating and inhibiting Fc-gamma receptors (5). However, virus-related mechanisms, including megakaryocyte infection; platelet surface alteration, accelerating their clearance; hypersplenism; hemophagocytosis; microangiopathic syndromes; and liver dysfunction, leading to reduced thrombopoietin production, may aggravate thrombocytopenia (5).

## Immunodeficiency and Autoimmunity (Green Box)

**Figure 1:**
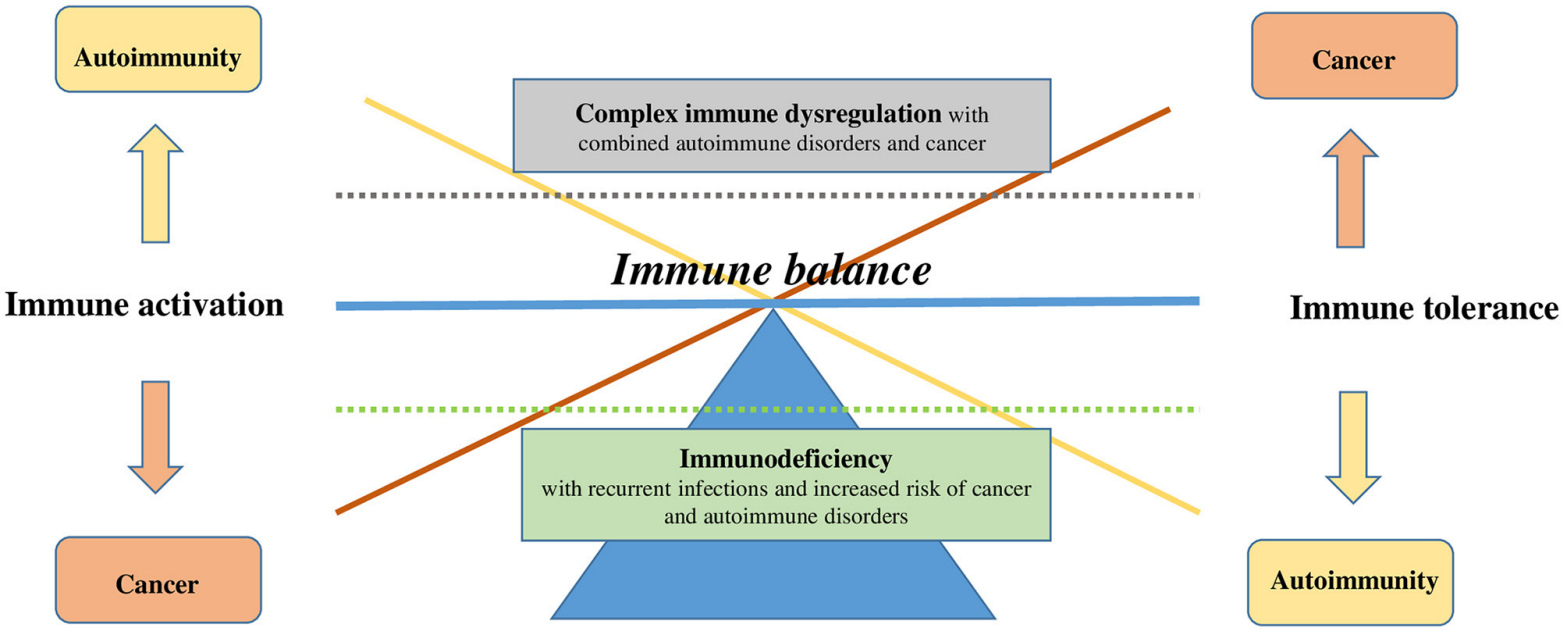
Dysregulation of immune balance. Immune balance can be disturbed on two levels: immune tolerance (on the right) or immune activation (on the left). Onset of a disease can be simplified by the shift in the balance on one side or the other (cancer in orange vs. autoimmunity in yellow) or on the vertical axis, represented by the dotted lines (immunodeficiency in green vs. complex immune dysregulation in gray).

Primary (genetically determined) and secondary (e.g., acquired by viral infections, drugs) dysfunction of the immune system have been linked to autoimmune disorders (Figure 1). Besides recurrent and opportunistic infections, the hallmarks of immunodeficiency are autoimmune cytopenia, such as ITP and autoimmune hemolytic anemia (17, 18). The clinician should therefore always consider the concomitant occurrence of autoimmune diseases and susceptibility to infections with caution. In the 40–50 s, the preventive and therapeutic administration of IgG concentrates (IVIG) resulted in a dramatic improvement of survival in numerous primary immunodeficiency diseases (PIDD) (19). In 1981, the immunomodulatory effect of IVIG in children with ITP (20) was observed to be the bridge to pathogenic and therapeutic challenges of the dysregulated immune system in autoimmune and immunodeficiency disorders. However, several disease associations are not well-understood, particularly the way of causality and recognition of the primary event.

**Figure 2:**
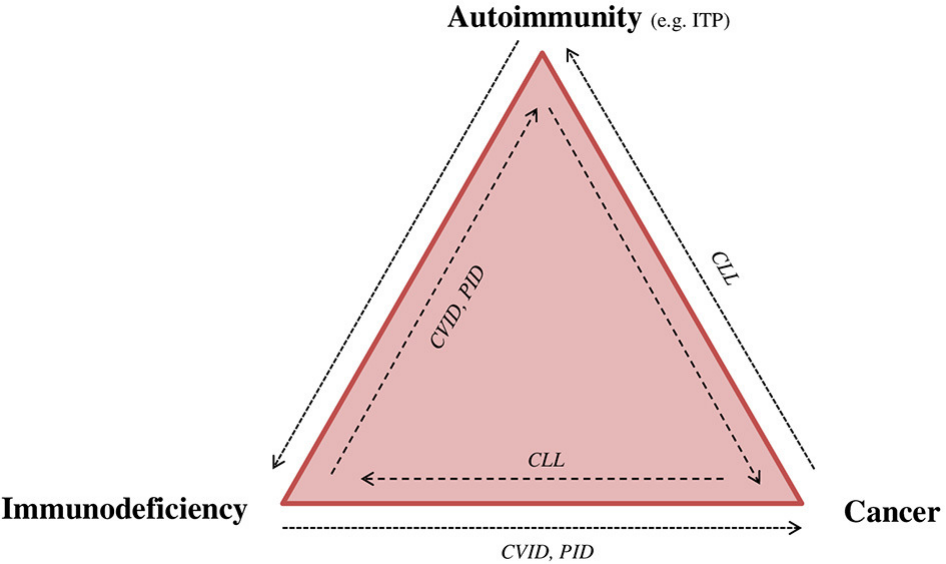
Immunodeficiency, autoimmunity, and cancer: “The immune trilogy.” Disturbance of the immune system can have multiple outcomes, such as cancer, autoimmunity or immune deficiency. The complexity and linkage between each of them can be demonstrated as “the immune trilogy.” Here are presented some examples of disease overlap. CLL, chronic lymphocytic leukemia; CVID, common variable immunodeficiency; ITP, immune thrombocytopenia; PID, primary immunodeficiency.

Patients with primary and secondary immunodeficiency have also an increased risk of certain cancers (Figure 2). For instance, PIDD patients have a significantly increased risk for lymphoma (10-fold in men and 8-fold in women) (21). Subsequent to infection, malignancy is the most prevalent cause of mortality in both PIDD children and adults. The type of malignancy depends on the immunodeficiency type, patient age, probably the occurrence of certain viral (e.g., HHV8, EBV) or bacterial infections (e.g., Helicobacter pylori), and the treatment administered (e.g., stem cell transplantation), indicating that different pathogenic mechanisms are implicated (22, 23). However, the concept of aberrant immune surveillance in immunodeficiency resumes the clinical duality (24).

## Cancer and Autoimmunity (Gray Box)

The first publication that reported the association between autoimmune disease and lymphoma was in 1966 (25). Subsequently, population studies have confirmed that certain autoimmune conditions, such as Sjögren syndrome, systemic lupus erythematosus, Hashimoto's disease, and rheumatoid arthritis, increase the risk of B-cell lymphoma (Figure 2) (26). Other reports have indicated a greater occurrence of T-cell lymphoma in celiac disease and ulcerative colitis patients (27). The frequency of lymphomas connected to an autoimmune disorder is estimated to be between 7% [e.g., Hodgkin's lymphoma (HL)] and 18% (e.g., marginal zone lymphoma) (28). Inversely, ITP occurs in the context of malignant lymphoproliferation (29), such as chronic lymphocytic leukemia (30), HL (31), and various types of non-Hodgkin's lymphomas (NHLs) (32, 33); it is rarely associated with T-cell NHL (29). The prevalence of ITP in HL has been estimated at 0.2–1% (31, 34), NHL at 0.76% (32), and chronic lymphocytic leukemia (CLL) at 1–5% (30). In a small case series, a high rate of lymphocytes with a CLL phenotype has been measured in autoimmune hemolytic anemia and ITP patients, assuming that some cases of ITP were probably initially misdiagnosed as primary forms (35). Cohort studies involving population-based nationwide databases showed an increased incidence for NHL and HL after the diagnosis of ITP, suggesting that ITP may also precede lymphoma onset (26, 36). However, the lymphoproliferative disease is generally considered to be the cause of thrombocytopenia, regardless of the diagnosis of cancer occurring months to years later. Several case reports have explained the causality of ITP and lymphoproliferation based on the assumption that treatments for lymphoma led to ITP resolution. In addition, tumor cells have been shown to produce antibodies against platelets or induce autoimmunity via antigen mimicry (37–39). In conclusion, whether ITP is a paraneoplastic phenomenon or an erroneous response of the immune system to the malignant cell remains unelucidated. Moreover, although it is unclear whether ITP may induce hematological malignancies, it could be possible, considering that the immunological process also occurs in the bone marrow, affecting stem cells.

The link between cancer and autoimmunity is the dysregulation of the immune system. T cells, B cells, and regulatory T and B cells as well as the innate cells (myeloid cells, dendritic cells) are involved in surveillance mechanisms of self and non-self (40–42). Many of the mechanisms that are considered essential in preventing autoimmunity suppress the immune response toward tumor cells (e.g., regulatory T cells) (40, 41, 43), placing cancer and autoimmunity in the opposite ends of the immune balance (Figure 1). Therefore, it appears quite remarkable that both conditions can co-occur, the reasons of which are probably multifactorial; further, the causality in one or another direction and the time scale are not always clear (44). The association of cancer and autoimmunity may also reveal an undiagnosed immunodeficiency syndrome.

For patients with autoimmune disorders, the risk of developing lymphoproliferative malignancies and solid tumors (45) is increased probably due to the combination of the underlying immune system dysregulation, immunosuppressive drug therapy, and chronic inflammation, thereby damaging tissue and further suppressing the immune system. Conversely, autoimmune disorders in cancer patients may appear as paraneoplastic phenomena or subsequent to immune therapies (e.g., checkpoint inhibitors) or as a second event based on the common genetic or environmental risk factors.

Although the pathogeneses of cancer and autoimmunity appear to be opposites, one common feature exists that supports the evolution of both diseases: chronic inflammation. Besides immune escape, cancer growth is well-known to be supported by a permissive tumor stroma that includes chronic inflammation and myeloid cells (46, 47). In autoimmune disorders, the dysregulation cascade becomes independent, facilitating the persistence and advancement of the disorder, regardless of the cause being removed, which can also be described as “chronic inflammation” and represents, in analogy to cancer, the microenvironment of autoimmune disorders (48).

## Therapeutic Analogies and New Developments

Therapeutic modalities in cancer have been a focus of research for several years. Targeted drugs and immunotherapy are currently integrated in numerous multimodal treatment strategies, complementing the classical cytostatic or cytotoxic approach (chemotherapy and radiotherapy). Currently, immunotherapy, such as monoclonal antibodies and T-cell therapies, is dramatically expanding. Nevertheless, challenges in the field remain tremendous. Targeting a tumor-specific antigen does not automatically lead to an antitumoral immunological response. The immunological activity (Th1/Th2 balance) may even be different for different epitopes of the same antigen.

In autoimmunity, therapies have particularly been focused on immunosuppression and immunomodulation (5). However, a long-term sustained response is usually limited. Reconstitution of tolerance in ITP has mostly yielded disappointing results, with only 30% of patients showing a sustained response following dexamethasone pulse therapy or rituximab therapy (49, 50). Curing autoimmune disorders is probably best achieved with treatments at an early stage of autoimmunity and by using a multimodal approach like that with anti-cancer treatments. Autoimmunity may evolve and become resistant to treatments with time and insufficient treatments (autoimmune expansion), similar to cancer. Novel approaches for the management of ITP with combined therapies against T and B cells and thrombopoietin-receptor agonists early in the course of the disease appear to exhibit promising results (e.g., mycofenolate mofetil/dexamethasone, rituximab/dexamethasone/eltrombopag, and rituximab/belimumab; ongoing studies include NCT03156452, NCT02834286, NCT03154385, NCT02760251, and NCT04812483) (51–53).

Cutting-edge treatments based on the knowledge of cancer and autoimmunity is emerging. New therapies that activate or reproduce autoimmune disorder pathways have been developed to target cancer, and reciprocally, immunological escape mechanisms of cancer have been investigated to treat autoimmune disorders. Strategies in suppressing or activating Tregs (54–56), dendritic cells (57), and myeloid-derived suppressor cells (58) are currently being studied. These cells are part of the tumor environment and support cancer growth, promote angiogenesis, and suppress immunological responses via different pathways (e.g., immunosuppressive cytokine expression and checkpoint inhibitors) (46, 59, 60). The role of myeloid-derived suppressor cells in autoimmunity is now being studied *in vitro*, which may result in novel therapeutic strategies (61).

## Conclusion

The pathogenesis of ITP is becoming increasingly understood, and secondary ITP is more likely identified. However, ITP remains to be a syndrome of complex immunological dysfunction. The clinical overlap between cancer, immunodeficiency, and autoimmune disorders exhibits an immunological activity that can simultaneously be overactive and suppressed or defective. Various endogenous (e.g., genetic predilection) and exogenous (e.g., infection) factors may trigger immune dysregulation with an intrinsic progression. Understanding analogies and bridges between cancer, immunodeficiency, and autoimmunity may help define driver immunological pathways, ultimately resulting in possible novel therapeutic strategies.

## Author Contributions

AS wrote the manuscript. FC, BG, HL, and MR presented the topic at the ICIS meeting and reviewed the manuscript. TK and PI reviewed the manuscript and provided critical feedback. AS, PI, and TK conceived the original idea. All authors contributed to the article and approved the submitted version.

## Conflict of Interest

AS: Amgen (research funds) and Novartis (advisory board). BG: served as an expert for Amgen, Novartis, Grifols, LFB, and Roche, and received grands for the research from Amgen. TK: Novartis (research funds), Amgen (research funds), and UCB biosciences (advisory board). The remaining authors declare that the research was conducted in the absence of any commercial or financial relationships that could be construed as a potential conflict of interest.
